# Ion Channels as Potential Tools for the Diagnosis, Prognosis, and Treatment of HPV-Associated Cancers

**DOI:** 10.3390/cells12101376

**Published:** 2023-05-12

**Authors:** Andrea Jazmín Chiliquinga, Brenda Acosta, Ingrid Ogonaga-Borja, Fernanda Villarruel-Melquiades, Jaime de la Garza, Patricio Gariglio, Rodolfo Ocádiz-Delgado, Ana Ramírez, Yesennia Sánchez-Pérez, Claudia M. García-Cuellar, Cecilia Bañuelos, Javier Camacho

**Affiliations:** 1 Grupo de Investigación de Ciencias en Red, Universidad Técnica del Norte, Ibarra 100105, Ecuador; 2 Departamento de Farmacología, Centro de Investigación y de Estudios Avanzados del Instituto Politécnico Nacional (CINVESTAV-IPN), Ciudad de Mexico CP 07360, Mexico; 3 Unidad de Oncología Torácica y Laboratorio de Medicina Personalizada, Instituto Nacional de Cancerología (INCan), Tlalpan, Ciudad de Mexico CP 14080, Mexico; 4 Departamento de Genética y Biología Molecular, Centro de Investigación y de Estudios Avanzados del Instituto Politécnico Nacional (CINVESTAV-IPN), Ciudad de Mexico CP 07360, Mexico; 5 Facultad de Ciencias Químicas e Ingeniería, Universidad Autónoma de Baja California, Calzada Universidad 14418, Tijuana 22390, Mexico; 6 Subdirección de Investigación Básica, Instituto Nacional de Cancerología (INCan), Tlalpan, Ciudad de Mexico CP 14080, Mexico; 7 Programa Transdisciplinario en Desarrollo Científico y Tecnológico para la Sociedad, Centro de Investigación y de Estudios Avanzados del Instituto Politécnico Nacional (CINVESTAV-IPN), Ciudad de Mexico CP 07360, Mexico

**Keywords:** HPV, cancer, ion channels, molecular markers

## Abstract

The human papilloma virus (HPV) group comprises approximately 200 genetic types that have a special affinity for epithelial tissues and can vary from producing benign symptoms to developing into complicated pathologies, such as cancer. The HPV replicative cycle affects various cellular and molecular processes, including DNA insertions and methylation and relevant pathways related to pRb and p53, as well as ion channel expression or function. Ion channels are responsible for the flow of ions across cell membranes and play very important roles in human physiology, including the regulation of ion homeostasis, electrical excitability, and cell signaling. However, when ion channel function or expression is altered, the channels can trigger a wide range of channelopathies, including cancer. In consequence, the up- or down-regulation of ion channels in cancer makes them attractive molecular markers for the diagnosis, prognosis, and treatment of the disease. Interestingly, the activity or expression of several ion channels is dysregulated in HPV-associated cancers. Here, we review the status of ion channels and their regulation in HPV-associated cancers and discuss the potential molecular mechanisms involved. Understanding the dynamics of ion channels in these cancers should help to improve early diagnosis, prognosis, and treatment in the benefit of HPV-associated cancer patients.

## 1. Introduction

The human papillomavirus (HPV) group comprises members with a high affinity to the cutaneous and mucosal epithelium. Around two hundred genetic types of HPV have been described and grouped into five genera (α, β, γ, μ, and ν), according to the variability of the viral capsid protein gene L1 [1,2].

The α group includes approximately 65 types with an affinity to mucosal epithelium and is commonly sexually transmitted. Its prevalence is high in teenage women and other young adults of active reproductive age, generally around 20 years, and its prevalence decreases in older adults [3]. Some HPV α-genetic types are non-dangerous or non-oncogenic related. For example, the HPV-62, HPV-89, HPV-11, and HPV-6 types are considered to be low-risk (LR-HPV) types and are associated with genital warts and papillomatosis, representing a prevalence of 11.6% [4,5]. On the other hand, the HPV-16, HPV-18, HPV-51, HPV-52, and HPV-53 types are reported as high-risk (HR-HPV) types, with a 13% prevalence, and are associated with malignant cytology and neoplastic processes [6]. Finally, a 5.2% prevalence corresponds to the HPV-53, HPV-66, HPV-70, and HPV-68 types, which are described as probably dangerous [5]. The β group includes around 50 HPV types with cutaneous epithelium affinity and mainly produces cutaneous lesions, while the γ, μ, and ν genera are considered benign. The most important high-risk types are HPV-16 and HPV-18 [6,7,8,9]^,^ as well as other types, depending on the studied population. For example, in Asian populations, cervical cancer has been associated with HPV-52 and HPV-58 types in addition to HPV-16 [10].

Other HPV-associated cancers include vulvar, vaginal [11], and anal cancer in women [12], while in men they include penile [13], testicular, and anal cancer [14,15]. The second HPV-associated cancer group is that of oropharyngeal cancers and involves head and neck [16,17,18], tongue, and laryngeal cancers [5,19]. Finally, other less frequent HPV-related cancers are lung [20], colon [21], bladder [22], prostate, and breast cancers [23,24]. The relationship between HPV genetic types and different types of cancers is shown in Table 1.

The tumorigenesis associated with high-risk HPV infections has been mainly related to the E5, E6, and E7 viral genes, whose corresponding oncoproteins are responsible for the altered regulation of several cellular pathways. Thus, E6 and E7 genotyping is currently used in some countries, including Finland [25], Canada [26], and Mexico [7], as a complementary test together with cytology for timely cervical cancer, neoplasms, and other HPV-related cancer diagnoses [26]. The inhibition of apoptosis, the induction of cell proliferation, immune system evasion, the promotion of metastasis, and the dysregulation of the cell cycle are some of the processes orchestrated by E5, E6, and E7 proteins [27]. Interestingly, the altered expression of some ion channels has been related to viral proteins [28], making certain ion channels predictive and therapeutic molecular markers for different cancer types [29,30,31,32].

## 2. HPV-Replicative Cycle Association with Cellular Pathways and Ion Channels

**Figure 1:**
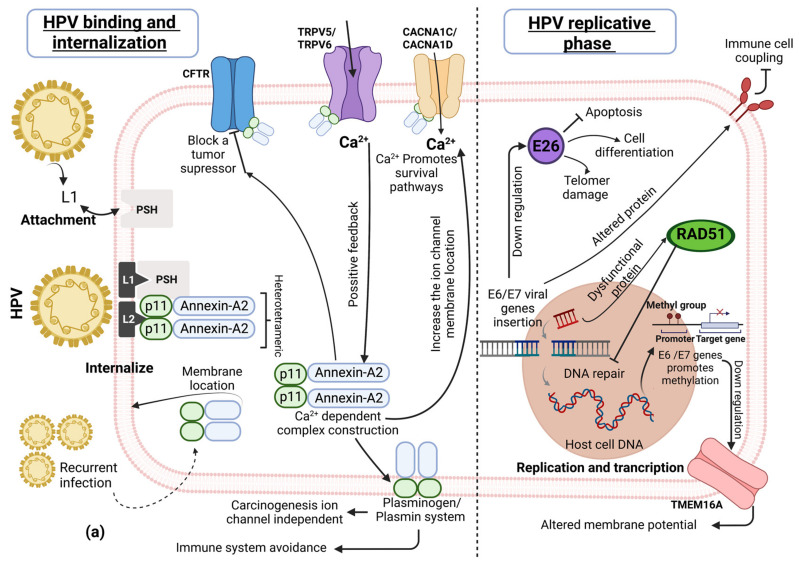

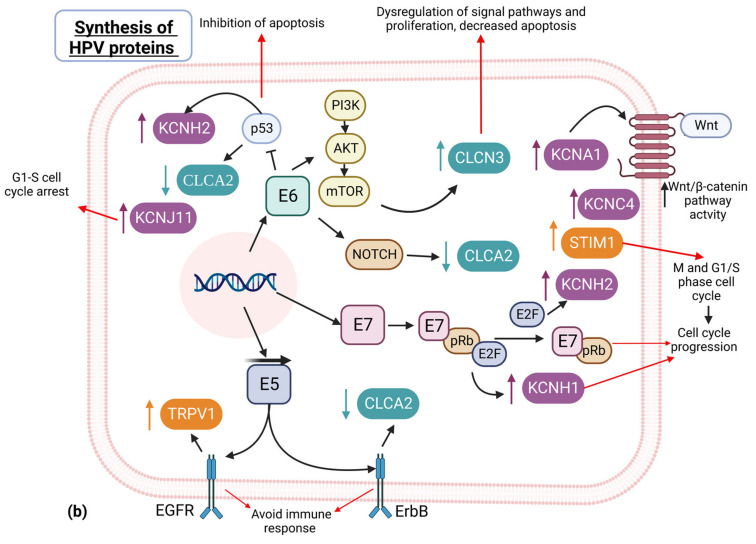
HPV-replicative cycle association with cellular pathways and ion channels. (**a**) HPV attachment and internalization is regulated by Annexin-A2/p11, which in turn affects calcium and chloride channels. Additionally, in HPV replication and transcription, the DNA insertion and DNA methylation compromises the DNA repair process by ion channel-dependent and -independent pathways associated with cell proliferation or tumor suppression. (**b**) HPV oncoproteins E5, E6 and E7 alter several pathways regulating some ion channels in cervical cancer.

We will first provide illustrative and general examples of how HPV infection modifies certain cellular pathways and ion channels associated with tumor progression. The high-risk HPV replicative cycle starts with the virus attachment to the host cell via L1 capsid viral protein coupling with the cellular heparin sulphate proteoglycans [33]. The HPV virus has a special affinity to active and proliferative cells, especially to basal epithelial cells, because the virus does not have DNA replicative enzymes and depends on the active cell cycle. The infection of the basal epithelia is successful due to epithelium micro-abrasion. Interestingly, it was recently found that the cervical transformation zone is very important in HPV infection [34]. Then, a secondary mechanism is activated to internalize the virus via the L2 capsid viral late protein and different cellular receptors, including epidermal growth factor receptors (EGFR), integrins, and annexin-A2. Thereafter, nuclear internalization takes place when HPV enters through nuclear pores, typically in the mitotic phase [2]. Interestingly, during the intracellular delivery of the virus, Annexin-A2 is initially a monomeric cytoplasmic protein, together with the p11 protein domain. Annexin-A2 and the p11 (S100A10) domain form a heterotetrametric complex, consisting of two Annexin-A2 units and a dimer of S100A10 [33,35]. This protein complex is translocated to the epithelial cell membrane due to tropism between the L2 HPV capsid protein and *S100A10* [36]. This information suggests that a persistent HPV infection stimulates the cell membrane Annexin-A2 protein complex location and ensures HPV’s infective success (Figure 1a). Some studies propose that the heterotetramer (Annexin-A2/S100A10) modulates ion channels and other membrane receptors [37,38].

CFTR, *TRPV5*, *TRPV6*, *CACNA1DC*, and *CACNA1D* ion channels are modulated by Annexin-A2/S100A10. The Annexin-A2 complex binds to TRPV5/6 (transient receptor potential vanilloid) ion channels, increases their cell surface location, and promotes the entry of calcium [38]. Calcium is necessary to stimulate heterotetrameric complex construction, thus promoting positive feedback [39]. TRPV channels and other calcium channels also modulate the intracellular calcium implicated in proliferation, apoptosis, and metastasis in different cancer types [40]. Particularly, *TRPV6* upregulation is involved in ovarian, prostate, pancreas, and breast adenocarcinoma [41]. Moreover, the CFTR (cystic fibrosis conductance regulator protein) channel facilitates the entry of chloride ions, is considered a tumor suppressor, and is involved in the proper epithelial differentiation. However, the Annexin-A2 complex affects chloride ion flux via CFTR [38], and could therefore lead to tumor development [42]. The Annexin-A2/S100A10 complex has itself been implicated in some cancer progression pathways that are dependent on the immune response and plasminogen production [37].

After virus attachment and internalization into the host cell nucleus, the HPV starts the replicative and transcriptional process. Initially, the virus transcribes and traduces the early E1 and E2 viral proteins [2,43]. Viral early proteins act as replication factors on E5, E6, and E7 genes to maintain and regulate the replicative cell cycle. The replication and transcription viral phases produce HPV copies using the viral episomal DNA as a template [44]. When the HPV infection is persistent, the E1 and E2 proteins also promote a level of viral DNA integration in the host chromosome, producing a particular methylation profile. *E6* and E7 viral genes could be integrated into hotspot genes, such as *FHIT*, *KLF5, LINC00392, RAD51B, CAS8, CASC21, ERBB2, TP63*, *MYC, CCND1*, and *CTTN* and promote somatic alterations [45], including transcriptional modifications on oncogenes, tumor suppressor genes, cell cycle regulators, and transcriptional factors [46].

The *ETS2* gene is located on chromosome 21 (encodes the E26 protein) and acts as a transcriptional factor and tumor suppressor but is affected by viral insertion [18]. HPV insertion could repress *ETS2* expression, compromising telomere integrity and possibly DNA damage accumulation. Another effect is the reduction in apoptosis, resulting in optimal conditions for HPV replication [47,48]. On the other hand, the *RAD51B* gene encodes the RAD51 protein, but the HPV genome integration produces a dysfunctional protein, altering the genome repair and inducing the accumulation of damage in the genome [18,49].

The *CD274* gene encoding the PDL1 protein is located on chromosome 9 and is affected by HPV insertion, finally producing an altered PDL1 transmembrane protein. This HPV replicative mechanism complicates the host’s immune response because altered PDL1 significantly reduces antigen recognition [45,50].

Gene hypermethylation by HPV has been observed in genes such as *BARX2* (a tumor suppressor) and *IRX4* (methylation sensible gene) [18]. Additionally, the transmembrane protein TMEM16A gene (which encodes the Ca^2+^-dependent chloride channel *ANO1*) is downregulated, affecting the cell membrane potential and promoting the neoplastic process, for example, in HPV+ head and neck carcinoma [28,51]. The specific methylation pattern produced by HPV may depend on the tissue and the persistent viral infection.

In the final HPV replicative cycle, E5, E6, and E7 oncoprotein expression have a key role in different cell pathways [44]. The E6 viral protein degrades the p53 protein (also known as “the guardian of the genome”) [52]. The consequence of p53 damage is the dysregulation of PI3K and AKT signal pathways, as well as cell proliferation and a decrease in apoptosis, among other effects. Interestingly, certain potassium channels are also dysregulated, potentially promoting cell cycle progression and the inhibition of apoptosis [53].

On the other hand, the tumor suppressor retinoblastoma protein (pRB) has important cell functions in the complex with the transcription factor E2F. pRB-E2F also regulates proteasomal protein degradation. The E7 protein sequesters pRB and promotes the detachment of E2F, leading to cell cycle progression [54]. Additionally, E7-pRb is more sensitive to ubiquitination and protein processing. The destabilization of pRb may be involved in the deregulation of the expression of some potassium channels, including *KCNH1* [55], *KCNC4* [56], and *KCNA1* [57], as well as calcium channels [58] (Figure 1b).

Another interesting example of the association between HPV and ion channels is the ATP-sensitive potassium (K_ATP_) channel, which favors the progression of the cell cycle and proliferation. E7 induces the expression of the SUR subunit of the channel, which in turn favors cell proliferation [59]. 

The E5 protein has a high affinity to receptors for important cytokines, such as EGF and ErbB. E5 works together with E7 to repress the gene expression of EGFR [60]. In addition, the *TRPV1* calcium channel promotes the ubiquitination of EGFR, modulating the EGFR/MAPK pathway [61,62].

In summary, several cellular proteins involved in the HPV-replicative cycle affect different pathways, leading to neoplastic cell transformation and including the participation of several ion channels. These transmembrane proteins have been suggested as important components of the hallmarks of cancer because their dysfunction is associated with cell proliferation, apoptosis, metastasis, angiogenesis, and immune system avoidance, among other processes [63].

Next, we describe and discuss the expression status and relationship of ion channels with different HPV-associated cancers and their potential uses as clinical markers. HPV infection is considered an etiological factor of some types of cancers, especially cervical and head and neck cancers. In certain other cases, including anal, penile, and vulvar cancers, a potential association with HPV has been suggested; however, the possible involvement of ion channels in these cancers has not been investigated in detail [64]. Thus, here we mainly focus on HPV-associated cancers as recognized by the International Agency on Research for Cancer (IARC), such as cervical and head and neck cancers. Nevertheless, we were also interested in reviewing ion channel expression in lung cancer because its potential association with HPV infection remains controversial [65]. Unfortunately, lung cancer is a malignancy with one of the worst mortality-to-incidence ratios worldwide. Therefore, the potential use of ion channels as clinical tools for this cancer may have benefits, although its association with HPV infection remains controversial. There is no doubt that this controversy may be resolved in some decades with patients receiving the vaccine against HPV.

A few examples of the potential association of HPV and ion channels in other cancers are also mentioned to provide the reader with a view of the broader impact of this topic in cancer. The present review focuses on ion channels and HPV-associated cancers. Several extraordinary publications have reviewed the association of ion channels and cancer, and some of these are cited in this review. However, we apologize to those authors whose contributions could not be cited.

## 3. Ion Channels as Potential Clinical Tools in HPV-Associated Cancers

Cancer is a multifactorial disease, and ion channels are regulated by a variety of cancer-associated factors, including virus infection, hormones, carcinogens present in cigarette smoke, air pollution, etc. Therefore, it is not easy to determine whether changes in ion channel expression are solely attributed to HPV infection [66]. However, when information is available, we can distinguish whether the analyzed cells or samples from patients were identified as HPV^+^ and whether the channel expression was compared with the corresponding normal cells or tissues. Interestingly, several of the studies here reported also analyzed ion channel expression status with the patients’ clinical outcomes.

### 3.1. Potassium Ion Channels

Potassium channels are found in all excitable and non-excitable cells [67]. They are responsible for the transport of K^+^ ions down its electrochemical gradient and play key roles in several processes, including the establishment of resting membrane potential, the regulation of action potentials, hormone secretion, and cell signaling [68,69]. In cancer cells, K^+^ channels have been implicated in cell proliferation, apoptosis, and migration [68]. In the following sections, the main groups of K^+^ ion channels studied and involved in carcinogenesis are discussed [68].

#### 3.1.1. Voltage-Gated Potassium Channels

Voltage-gated K^+^ (Kv) channels regulate cell excitability in response to changes in the cell membrane potential. Kv channels become activated with membrane depolarization and closed when the cell membrane is hyperpolarized [67]. The voltage sensing domain (VSD) movement is associated with pore opening [70]. Cancer cells undergo hyperpolarization upon exiting the G_1_ phase of the cell cycle, leading to the cell proliferation processes [71]. 

**Figure 2:**
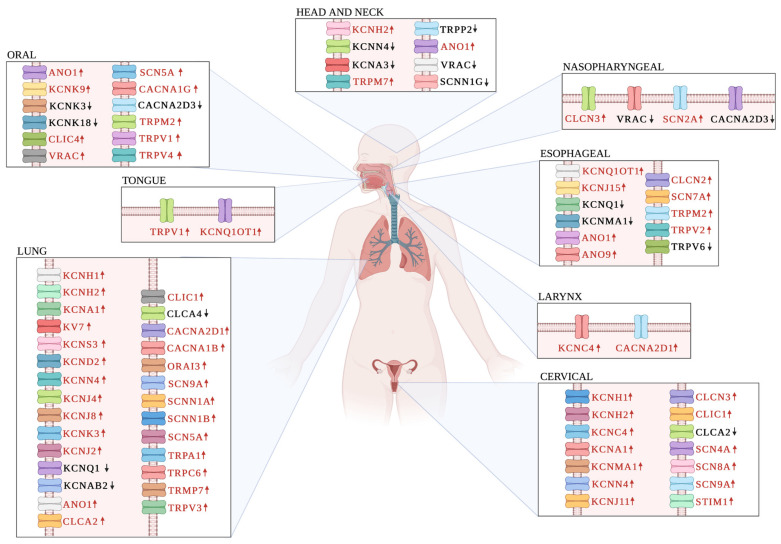
Expression status of ion channels in HPV-associated cancers. The figure illustrates the tissue location of a variety of ion channels involved in carcinogenesis of HPV-associated cancers in both sexes. Ion channel overexpression is represented in red letters and up-arrow, while letters in black and down-arrow indicates downregulated ion channel expression. Together, these observations suggest a strong oncogenic or tumor suppressor role for many ion channels in HPV-associated cancers.

K_V_10.1 (*KCNH1*, Ether-à-go-go 1 (Eag1)) is expressed in a few normal tissues, including the brain, adrenal glands, placenta, and testis; however, in cancer tissues its expression is more ubiquitous and abundant [71]. Interestingly, the level of this channel is negatively regulated by retinoblastoma and the E2F pathway [55]. High *KCNH1* expression has been demonstrated in several biological models of lung and HPV+ cervical cancers (Figure 2) [32,72]. *KCNH1* has been proposed as a therapeutic target by using certain channel blockers (Table 2). For example, astemizole is a second-generation antihistamine that blocks some K^+^ channels, including *KCNH1* and *KCNH2* [73], and reduces the proliferation of HPV+ cervical cancer (CC) cells [74], while in non-small cell lung cancer (NSCLC) cell lines, this drug did not prevent the epithelial-to-mesenchymal transition (EMT) despite the fact that *KCNH1* gene and protein expression were up-regulated during EMT [72]. These results suggest that the role of *KCNH1* channels in EMT is not solely related to their conductive functions [72].

The human Ether-à-go-go-related gene Kv11.1 (*KCNH2* or hERG1) is expressed in several normal tissues, including cardiac tissue and smooth muscle, and in cancer cells it is regulated by several microRNAs relevant to tumor progression [53]. The overexpression of the channel in different head and neck cancers is associated with cell growth, invasiveness, malignant transformation, and poor prognosis [75,109,124,174]; likewise, in HPV+ cervical adenocarcinoma, the inhibition of hERG1 alters cell cycle development [125]; meanwhile, in lung cancer, the gene silencing and application of channel blockers decreases cell proliferation (Table 2) [175].

Kv3 channels participate in cancer cell proliferation, migration, and metastasis via the AKT signaling pathway and vimentin, which is involved in cell migration and EMT [56]. In CC HPV+ cell lines, the potassium voltage-gated channel subfamily C member 4 (*KCNC4* or Kv3.4) and the AKT pathway have been shown to regulate vimentin expression, and the use of blood depressing substance II (BDS-II) as a channel blocker (Table 2) decreases vimentin expression and cancer cell migration [56]. Similarly, in head and neck cancer, a high expression of *KCNC4* is associated with laryngeal and pharyngeal squamous cell carcinoma; in accordance, the silencing of *KCNC4* by siRNA inhibits cell proliferation in the G_2_/M phase [121]. The transcriptional factor HIF-1α regulates cell migration and proliferation through *KCNC4* channels [108].

The potassium voltage-gated channel subfamily A member 1 (*KCNA1* or Kv1.1) plays an important role in the control of neuronal excitability [122]. In CC HPV+, *KCNA1* overexpression in samples from patients is associated with the poor prognosis of the disease, the induction of mitochondrial dysfunction in cell lines, and the regulation of the Hhg, Wnt, and Notch signaling pathways, while *KCNA1* silencing arrests cell growth, invasion, and migration, as well as improving survival time, in nude mice [57]. In non-small cell lung cancer, the application of the *KCNA1* blocker dendrotoxin-_K_ (DTX-_K_) reduces cell viability and tumor size in mice [126].

Kv1.3 (potassium voltage-gated channel subfamily A member 3, *KCNA3*) channels regulate membrane potential and Ca^2+^ entry in human effector memory T cells (T_EM_). In head and neck cancer, tumor infiltrating lymphocytes (TILs; cells that can be recruited by the tumor cells and the cytokines that they produce can help tumor cell survival) display up to a 70% reduction in functional *KCNA3* channels, consequently decreasing Ca^2+^ entry and CD8 T cell function (Figure 2) [107].

On the other hand, a differential expression of KCNQ channels has been observed in lung cancer, with higher expressions in side-populations than in main-population cells; moreover, when side-population cells were treated with the EGFR antagonist gefitinib [176] in combination with KCNQ blockers (TEA, 4-AP) or openers (flupirtine), the gefitinib resistance decreased [76]. The deletion of potassium voltage-gated channel modifier subfamily S member 3 (*KCNS3* or Kv9.3) in lung adenocarcinoma (LUAD) cell lines inhibits cell proliferation by cell cycle arrest in the G_0_/G_1_ phase (Table 2) [77]. Additionally, potassium voltage-gated channel subfamily D member 2 (*KCND2* or Kv4.2) is deregulated in several tumors and its high expression in LUAD is associated with unfavorable clinical outcomes for patients [106].

Furthermore, Kv subfamily Q member 1 (*KCNQ1*) plays important oncogenic roles, mainly in cell cycle, apoptosis, and autophagy [127]. In esophageal squamous cell carcinoma (ESCC), miR-483e5p overexpression silences *KCNQ1* promoting cell proliferation, migration, and invasion [78]. Likewise, in lung cancer, *KCNQ1* overexpression has been associated with longer survival times for patients with LUAD [177]. Interestingly, the long non-coding RNA (lncRNA) *KCNQ1* overlapping transcript 1 (*KCNQ1OT1*) in tongue cancer promotes cisplatin resistance through the action of miR-124-3p, whereas in esophageal cancer, its overexpression is associated with worse prognoses via miR-133b [128,178]. *KCNQ1OT1* is overexpressed in lung adenocarcinoma and is associated with resistance to stereotactic body radiotherapy [127] and its silencing reduces the expression of the multidrug resistance gene 1 (*MDR1*) in lung cancer cell lines (Table 2) [129].

Kv subfamily A regulatory beta subunit 2 (*KCNAB2*) participates in neuroendocrine functions as well as in certain cancer processes [105]. Its low expression in LUAD is associated with accelerated tumor growth and poor prognosis, whereas its overexpression contributes to the expression of chemokines required for immune cell migration [104].

#### 3.1.2. Calcium-Activated Potassium Channels

Calcium-activated potassium channels are involved in neurosecretion, action potential formation, and the overall regulation of neuronal excitability, among other activities [179]. The up-regulation of the calcium-activated potassium channel, subfamily M, and alpha member 1 (*KCNMA1* or KCa1.1) in combination with verapamil (a drug used in the treatment of cardiac arrhythmias and the inhibition of transmembrane Ca^2+^ flux) [180] potentiates the cytotoxic effect of cisplatin against ESCC (Table 2) [130]. It is of interest that, in transgenic mice expressing the HPV16-E7 oncogene and treated with estradiol to induce cervical neoplasms and cancer, the *KCNMA1* gene and protein expression was increased, as well as in samples from patients with cervical lesions [29].

Calcium-activated potassium channel subfamily N member 4 (*KCNN4* or KCa3.1) participates in the activation of microglia, but it also influences migration, cell proliferation, activation, and cytokine release from blood cells and has been associated with apoptosis, metastasis, and drug resistance in cancer [181]. In head and neck cancer, the inhibition of programmed death receptor alpha 1 (αPD-1) increases the fluxes of *KCNN4* and Kv1.3 channel activity, thus improving the immune function [131]. Likewise, pembrolizumab, which is a humanized monoclonal antibody with high specificity towards PD-1, has been used in the treatment of several malignancies, including head and neck cancer [182], and increases *KCNN4* channel activity and CD8 T-cell chemotaxis (Table 2) [132].

In CC, *KCNN4* activation increases the sensitivity of cells to Hoechst 33258 dye, thus improving the penetration of cytotoxic substances into cancer cells [133]. *KCNN4* overexpression is associated with cell proliferation and reduced apoptosis in HPV+ CC cell lines; accordingly, clotrimazole together with *KCNN4* silencing decreases channel currents and cell proliferation [79]. In lung cancer, the overexpression of *KCNN4* is associated with proliferation, migration, invasion, tumorigenicity, and poor prognosis (Figure 2) via membrane potential hyperpolarization and the P13/AKT and MEK/ERK signaling pathways [103,183]. In addition, in non-small cell lung cancer, *KCNN4* channel blocking improves the response to the EGFR inhibitor gefitinib [184].

#### 3.1.3. Inwardly Rectifying Potassium Channels

Inwardly rectifying potassium (Kir) channels are responsible for the regulation of the resting membrane potential, thus modulating electrical activity, neuronal activity, insulin secretion, and K^+^ transport [185]. Kir channel subfamily J member 15 (*KCNJ15* or Kir4.2) is part of the Kir transport channels, and its inhibition in ESCC arrests cell proliferation, migration, and invasion, whereas its high expression is associated with shorter life expectancy compared to lower expression groups [80].

Kir channel subfamily J member 2 (*KCNJ2* or Kir2.1) and Kir channel subfamily J member 4 (*KCNJ4* or Kir2.3) have been involved in lung cancer. *KCNJ2* is responsible for maintaining the resting membrane potential and the regulation of cell excitability, although changes in its expression are associated with apoptosis, proliferation, and cell adhesion [186]. In small cell lung cancer (SCLC), *KCNJ4* modulates cell growth, and its silencing sensitizes lung cancer cell lines to the cytotoxic effects of adriamycin, cisplatin, and etoposide (Table 2) [134]. *KCNJ4* is up-regulated by EGFR-associated pathways [81], and *KCNJ4* overexpression in LUAD is associated with poor prognosis [102].

Finally, ATP-sensitive Kir channels play a role in coupling membrane potential to cell metabolism [187]. These channels consist of a K^+^ channel subunit forming the channel pore (Kir6.x) and a sulfonylurea receptor (SUR) [82], and have been associated with oncogenic processes in lung and HPV+ cervical cancers. Kir6.2 (*KCNJ11*), SUR1, and SUR2 are overexpressed in the cell lines and biopsies of patients with CC; in addition, when treating the cell lines with glibenclamide (a drug used in the treatment of diabetes that blocks *KCNJ11*) [188], cell proliferation was inhibited (Table 2) [82]. In NSCLC, the overexpression of SUR1 (*KCNJ8* or Kir6.1) promotes cell proliferation, and silencing *KCNJ8* or glibenclamide treatment decreases cell and tumor growth, as well as increasing expression of the tumor suppressor Krüppel-like factor 4 (KLF4) [83].

#### 3.1.4. Two-Pore Domain Potassium Channels

Two-pore domain potassium channels are involved in the regulation of physical, chemical, and mechanical stimuli and associated with several cell signaling pathways [189]. In oral squamous cell carcinoma (OSCC), the protein expression of *KCNK3* (TASK1 or K2P3) and *KCNK18* (TRESK or K2P18), which belong to the family of tandem pore domains in weakly inward rectifying K^+^ (TWIK) channels, was found to be decreased in rat tumors and cancer patient tissues compared to normal samples, unlike *KCNK9* (TASK3 or K2P9), which was found to be overexpressed in the OSCC samples compared to normal tissue [84]. However, *KCNK3* are overexpressed in NSCLC cell lines and their silencing reduces proliferation and enhances apoptosis [135].

### 3.2. Sodium Ion Channels

Sodium channels are transmembrane proteins that are highly selective for Na^+^ transport in favor of its electrochemical gradient. Among the main functions of these channels are the generation and propagation of action potentials and the regulation of neuronal excitability and ion transport [190]. There are two main families of these channels: voltage-gated sodium channels (VGSC, Nav) and epithelial sodium channels (ENaC) [191,192]. These two types of channels are aberrantly expressed in cancer cells and involved in proliferative processes [193].

#### 3.2.1. Voltage-Activated Sodium Channels

Voltage-gated sodium channels play essential roles in the electrical activity of excitable cells, including the generation and propagation of action potentials in neurons and neuroendocrine cells, as well as cardiac, skeletal, and smooth muscle [194]. Nine subtypes of VGSC (Nav1.1–Nav1.9) have been characterized and are encoded by the *SCN1A-SCN11A* genes and are highly conserved in humans [195]. Structurally, they are formed by a complex of an α-subunit associated with one or more β-subunits, and although these channels have similar functions, some differences in their properties and isoforms determine their role in the physiology of some diseases [196,197]. The activation of sodium channels takes place after membrane depolarization, and the resulting sodium entry further depolarizes the membrane, generating action potentials [197,198].

Voltage-regulated sodium channels increase the motility and invasiveness of cancer cells, depending on the tissue in which they are expressed; therefore, understanding their regulation in cancer development can lead to innovative therapies and molecular biomarkers for several cancers [199]. The *SCN5A* (Nav1.5) channel has been studied in the OSCC cell line SCC-15 and tissues of patients. The up-regulation of this channel in OSCC is associated with proliferation, migration, and invasion and correlates with metastasis in lymph nodes as well as with high neutrophil-to-lymphocyte ratio (NLR) and platelet-to-lymphocyte ratio (PLR), which are indicators of the inflammatory state of the organism [114,145].

Interestingly, the expression of Nav1.5 is induced by EGF in OSCC HSC-3 cells and channel expression is associated with cell proliferation and migration via the Wnt/β-catenin signaling pathway [146]. In addition, the inhibition of Nav1.5 channel activity with ranolazine reduces breast cancer metastasis and pulmonary colonization [147].

The voltage-regulated sodium channel *SCN8A* (Nav1.6) has been studied in tissue samples from HPV16+ CC patients, primary cultures, and cell lines. HeLa (HPV18+), SiHa, and Caski (both HPV16+) cells show the overexpression of *SCN8A*, and its induced expression increases the activity of the matrix metalloproteinase MMP-2, which leads to cancer invasion and progression [148,200] (Table 1). The inhibition of *SCN8A* in primary cultured CC cells using Cn2 toxin and tetrodotoxin significantly reduces the invasiveness of these cancer cells [93].

Concerning the voltage-regulated sodium channel *SCN7A* (Nav2.1), an in silico study in ESCC showed mutational signatures in patient tumors which were associated with tumor vascular invasion and shorter survival time, suggesting that *SCN7A* has a high value as a prognostic marker for patients with ESCC [92]. On the other hand, a study performed in NSCLC reported that the functional expression of *SCN9A* (Nav1.7) is controlled by EGF/EGFR signaling through the ERK1/2 pathway, which in turn promotes the invasion of H460 lung cancer cells.

#### 3.2.2. Epithelial Sodium Channels

Epithelial sodium channels (ENaC) are passive transport ion channels present in the apical membrane of epithelial cells [123]. They are formed by three homologous subunits (α, β, and γ) that form the pore, allowing the movement of Na^+^ from the extracellular fluid to the cytoplasm in numerous re-absorptive epithelia, such as the distal part of the renal tube, lungs, the respiratory tract, the female and male reproductive tracts, placenta, and colon, among others [123,201]. Na^+^ reabsorption is regulated by hormones, such as aldosterone, vasopressin, and glucocorticoids; among the main functions mediated by ENaC are electrolyte homeostasis, the regulation of extracellular fluid volume, and arterial pressure [202].

The malfunction of ENaC because of deletions or loss-of-function mutations in any of its subunits leads to its over- or under-expression, which in turn is related to several human diseases, such as the Liddle syndrome, cystic fibrosis, multisystemic pseudo-hypoaldosteronism, and essential hypertension [123]. Its deregulation is also involved in tumor development and progression, for example, the ENaC alpha subunit mediates cancer cell proliferation and migration processes [149]. Thus, ENaCs are increasingly being studied as potential therapeutic targets and diagnostic and prognostic markers in cancer [203].

The *SCNN1B* gene encodes for the beta (β) subunit of ENaC, and bioinformatic analysis identified six candidate genes as biomarkers in LUAD, suggesting that the hypermethylation of *SCNN1B* significantly decreases its expression. This was associated with the short survival of patients, making the channel a prognostic molecular biomarker [203]. Further studies of the SCLC cell line H889 found that the *SCNN1A* gene encoding the α-subunit of ENaC directly correlates with the expression of ASCL1, which is essential in cancer cell progression and survival, for example, in a subset of lung cancer [204].

In addition, the *SCNN1G* gene coding for the γ subunit of ENaC has been studied in head and neck squamous cell carcinoma, where it is downregulated, and this correlates with tumor metastasis and poor prognosis in HNSCC patients, suggesting a tumor suppressor role. Moreover, the function of *SCNN1G* has been analyzed under overexpression conditions, where γENaC inhibits HNSCC cell migration by increasing adhesive strength and cell–cell integrity, thus highlighting its potential as a diagnostic biomarker in HNSCC. However, its use as a biomarker is limited to certain types of cancer, as other studies have reported the activity of γENaC as a tumor promoter in breast cancer [113,205] (Figure 2).

### 3.3. Chloride Ion Channels

Chloride channels (Cl^−^) are ubiquitously expressed in all cell types [206]. These ion channels are found in the plasma membrane and intracellular organelles, where they serve vital functions, including the regulation of ionic homeostasis, membrane excitability, intracellular pH, transepithelial transport, proliferation, and cell volume [207,208]. Cl^−^ flux across the plasma membrane regulates neuronal and muscle excitability by changing the membrane potential [209]. Because of their important role in the maintenance of cellular homeostasis, mutations in genes encoding these channels can trigger the development of human pathologies, such as myotonia, epilepsy, cystic fibrosis, and cancer cell migration and infiltration [210]. The families of chloride channels that have been implicated in cancer are the voltage-dependent chloride channels (VDC), calcium-activated chloride channels (CaCl), volume-regulated chloride channels (VRAC), and chloride intracellular channels (CLIC).

#### 3.3.1. Voltage-Dependent Chloride Channels

Voltage-dependent chloride channels (CLC) mediate Cl^−^ flux in favor of their electrochemical gradient. Their main function is to establish the resting membrane potential and chloride concentration in intracellular compartments. There are nine subtypes of the CLC channel family, where *CLCN3* (CLC-3) expression is related to cell cycle regulation and cell proliferation, migration, and apoptosis [211,212] (Figure 2). In cervical squamous cell carcinoma, the *CLCN3* channel is involved in cell invasion and migration processes and includes the participation of the PI3K/AKT/mTOR signaling pathway [94]. 

Studies performed in nasopharyngeal carcinoma cells (CNE-2Z) indicate that the selective activation of *CLCN3* chloride ion channels is closely related to apoptotic events [213]. The expression of the proapoptotic proteins caspase-3 and BAX was upregulated in zolendronic acid (ZA)-treated cells via the production of reactive oxygen species (ROS), which in turn activated the *CLCN3* channel, triggering cell apoptosis [120,211] (Figure 2). Similarly, dihydroartemisinin (DHA) treatment in CNE-2Z cells increased the expression of *CLCN3* chloride channels and enhanced their activity. The increase in Cl^−^ currents induced apoptotic volume depletion (AVD) and the activation of caspase-3 [173]. Interestingly, the inhibition of this channel at specific stages of the cell cycle was able to arrest cancer cell proliferation; however, when it is persistently expressed with the help of activators, it leads to cell apoptosis. Thus, its importance as a possible therapeutic target in cancer is highlighted [214,215].

The voltage-dependent chloride channel *CLCN2* (CIC-2) is involved in the maintenance of membrane potential, transepithelial transport, the regulation of cell volume, and cell proliferation and survival [216]. The lubiprostone-mediated activation and overexpression of *CLCN2* in ESCC reduces cancer cell proliferation by acting as a tumor suppressor via the IFN signaling pathway [95]. 

#### 3.3.2. Calcium-Activated Chloride Channels

Calcium-activated chloride channels (CaCl) are present in excitable and non-excitable cells and become activated when cytosolic Ca^2+^ is increased [217]. These channels play important roles in different processes, including afterdepolarization, transepithelial transport, the regulation of cellular excitability, vascular tone control, epithelial secretion, and cell proliferation in carcinogenesis processes [218,219]. The depletion of Anoctamin-9 chloride channel *ANO9* (TMEM16J) by siRNA in KYSE150 and KYSE790 cells increased the number of cells under arrest in the G_0_/G_1_ phase, reducing cell proliferation and migration [119] (Figure 2).

The *ANO1* channel (TMEM16A) is involved in cancer cell proliferation, invasion, survival, and apoptosis; the signaling pathways that can be regulated by *ANO1* include ERK, PI3K-Akt, TNF, Ca-M, and EGFR [118,220,221]. Studies performed in head and neck squamous cell carcinoma (HNSCC) indicate that a high expression of *ANO1* is related to metastatic processes in lymph nodes, poor prognosis, and the poor survival of patients with OSCC and ESCC [96,97,171,172]. It is also suggested that *ANO1* directly correlates with the activation of the Erk½ pathway and increases tumor size and downregulates the expression of the proapoptotic protein Bim [98,99]. The overexpression of *ANO1* in HNSCC has pointed to its development as a potential prognostic, diagnostic, and therapeutic target [170,222] (Table 2). Some *ANO1* channel regulators, including fluoxetine [100] and casein kinase 2 (CK2), reduce cell migration activity and Notch signaling [117,169]. In addition, the combination of cisplatin with chelating agents, such as cuprizone, could produce a synergistic effect in the treatment of HNSCC overexpressing this channel [167,168].

ANO1 is also overexpressed in NSCLC tissues [220,223]. This overexpression and its association with clinical outcome (Figure 2) could be related to the expression and activity of EGFR [224,225]. Additionally, a study showed how the anticancer effect of verteporfin significantly reduces *ANO1* protein levels and EGFR-STAT3 activation in PC9 cells [166]. Growing evidence shows that the inhibition of *ANO1* expression or activity can serve as therapeutic agents in NSCLC and include matrine [165], homoharringtonine (HHT), and arctigenin [163,164] (Figure 2). Other therapeutic agents whose main effect is to decrease channel activity and the carcinogenic processes involved include benzophenanthridine, theaflavin, cepharantin, zafirlukast, and a hydrogel loaded with limonin [160,161,162,226,227].

#### 3.3.3. Volume-Regulated Chloride Channels (VRAC)

Cell volume is finely regulated by several mechanisms [228]. After cell swelling caused by increased intracellular osmolytes, the cell restores its volume to normal conditions. This process is called regulatory volume depletion (RVD) and occurs through the loss of K^+^, Cl^−^, and organic osmolytes [229,230]. The Cl^−^ conductance is critical for RVD and is mediated by the volume-regulated anion channel (VRAC)/organic osmolyte channel (VSOAC), also known as VSOR [231].

VRAC has been reported to regulate carcinogenic processes. When the cell is in isovolumetric conditions, the activation of VRAC causes cell shrinkage, cell contraction, or a decrease in apoptotic volume (AVD), a condition that mediates apoptosis in several cells [232]. During AVD, the Cl^−^ conductance increases, the cell depolarizes, and K^+^ is lost, thereby activating Caspase-3 and favoring cell apoptosis [233]. Apoptotic resistance in cancer cells involves the functional impairment of chloride channels, where decreased VRAC activity reduces cell contraction, inhibits Caspase-3 activity, and prevents cell apoptosis [234,235].

The treatment of head and neck cancers can be affected by the development of chemoresistance to cisplatin, the first-line treatment in patients with nasopharyngeal carcinoma [236]. A study based on the bioinformatics analysis of positive HPV tumor samples identified that the high expression of VRAC correlates to the effectiveness of cisplatin treatment, and in vitro assays in resistant cells show that reduced VRAC expression reduces cisplatin uptake and favors cancer cell survival [101]. Moreover, another study confirmed that the mechanism by which cisplatin exerts its function in CNE-2Z cancer cells (a human nasopharyngeal carcinoma cell line) is via the P2Y receptor, which induces the activation of VRAC and AVD, ultimately leading to cell apoptosis [159]. While in OSCC the VRAC-specific inhibitor DCPIB significantly decreased the proliferation of HST-1 cells, in other cancer cell types, VRAC may not be relevant for cell proliferation [158,237]. At any rate, VRAC is a possible therapeutic target and prognostic marker in drug resistance for certain HPV-associated cancers [159].

#### 3.3.4. Chloride Intracellular Channels (CLICs)

Intracellular chloride channel proteins are predominantly found in intracellular organelles [238]. These proteins function as both enzymes or channels, depending on the form in which they are found, either as soluble proteins or integral membrane proteins [239]. CLICs are a family of multifunctional proteins that can have diverse functions, including chloride transport or the regulation and modulation of gene expression or cytoskeleton structure [206,240]. Intracellular chloride channels are also involved in neurological, pulmonary, and cardiovascular functions and are related to pathologies such as cancer, as they regulate cell cycle and apoptosis [238,241,242].

The intracellular chloride channels *CLIC1* and *CLIC4* are promising cancer therapeutic targets because of their activities as ion channels and signal transducers in cell cycle progression and the malignant transformation of cancer cells [239]. The expression of CLIC chloride channels in the cell membrane may lead to tumor progression [243] by interfering with different signaling pathways [244] (Figure 1b).

*CLIC1* (CLCNL1) can be found either as a cytosolic or transmembrane protein affecting cell cycle progression and the migration and invasion of solid tumors; however, in reality, its overexpression in several cancers has led to the suggestion that it can act as a prognostic marker and therapeutic target [240]. For example, *CLIC1* shows increased expression in cell lines and tissue samples from HPV+ CC patients and has been proposed as a tumor promoter [153]. In a similar manner, *CLIC1* is upregulated in LUAD and is associated with tumor metastasis and shorter survival [245]. Furthermore, the inhibition of *CLIC1* in lung cancer cell lines decreases cell proliferation via the suppression of the p38 MAPK pathway, increased apoptosis, and the activation of Jun N-terminal kinases (JNK) [246,247]. This role converts this channel into a potential therapy target for this type of cancer (Figure 2).

CLIC4 (*CLIC4L*), in turn, fulfills functions in ionic homeostasis, transepithelial transport, and cell volume balance [248,249]. This channel is regulated by p53 and tumor necrosis factor-α (TNF-α), two relevant proteins in cancer [244]. Experiments performed in the squamous cell carcinoma of the lower lip (LLSCC) showed differential *CLIC4* expression and function depending on the stages of the disease [157,250]. A proteomic study in lung cancer revealed that the down-regulation of *CLIC4* is associated with carcinogenesis in some types of lung cancer. *CLIC4* restoration in lung cancer cell lines attenuates cell proliferation, suggesting *CLIC4* as a tumor suppressor [248,251].

### 3.4. Calcium Ion Channels

Calcium (Ca^2+^) regulates several physiological processes, including muscle contraction, transmitter release, cell differentiation and proliferation, gene transcription, and apoptosis [252]. Ca^2+^-permeable channels regulate Ca^2+^ concentrations in mitochondria, endoplasmic reticulum, lysosomes, and cytosol, with subsequent effects on cell proliferation, apoptosis, and autophagy that can trigger oncogenic processes [252]. Many Ca^2+^ channels are involved in carcinogenesis and will be discussed next. 

#### 3.4.1. Voltage-Gated Calcium Channels

Voltage-dependent calcium channels (Cav) play important roles in the regulation of mitosis, cell proliferation, and apoptosis [253]. Cav channel Alpha 2 delta 3 (*CACNA2D3*) is downregulated in nasopharyngeal carcinoma tissue samples and cell lines; when restoring its expression level, it influences the non-canonical Wnt/Ca^2+^ signaling pathway and induces apoptosis [156]. These reports are related to research in ESCC, where *CACNA2D3* is downregulated in cancerous samples, possibly due to DNA copy number loss with promoter hypermethylation, and thus, *CACNA2D3* is able to inhibit cell proliferation as well as colony and tumor formation in mice, possibly by increasing the expression of p53 and p21 [136].

Cav channel alpha 2 delta 1 (*CACNA2D1*) has been associated with cell proliferation [254]. *CACNA2D1* protein (α2δ1) has been implicated in several types of cancer, including laryngeal squamous cell carcinoma, where its expression is associated with miR-107 expression. When miR-107 is downregulated, α2δ1 is overexpressed, favoring the progression and poor prognoses of this pathology (Table 2) [254]. In contrast, α2δ1 contributes to the drug resistance of SCLC and participates in cell proliferation, invasion, and metastasis [255]. Likewise, in non-small cell lung cancer, the inhibition of α2δ1 with the mAb1B50-1 blocker delays the tumorigenicity of tumor-initiating cells [256].

The voltage-gated calcium channel subunit alpha 1B (*CACNA1B* or Cav2.2) is involved in the release of neurotransmitters that are mainly related to pain signaling in the central and peripheral nervous system [257]. In NSCLC, *CACNA1B* gene expression is higher in tumor tissue than in normal samples, which is associated with increased intracellular Ca^2+^ concentration, leading to cell proliferation [110].

Cav channel subunit Alpha 1G channel (*CACNA1G* or Cav3.1) mediates Ca^2+^ entry into excitable cells and is involved in muscle contraction, hormone and neurotransmitter regulation, and cell division [258]. In human squamous cell carcinoma, its expression was significantly higher than in normal mucosa and dysplasia. This overexpression is associated with the proliferation and inhibition of apoptosis, since its silencing by siRNA decreases these processes [87]. In lung adenocarcinoma, *CACNA1G* expression is different in the cell lines evaluated [88]. The T-type alpha 1H subunit (*CACNA1H* or Cav3.2) channels play important roles in chronic pain [259]. *CACNA1H* is expressed in LUAD cell lines and channel blockage inhibits cancer properties [88].

#### 3.4.2. Calcium Channels Activated by Calcium Release

Calcium release-activated calcium channels (CRACs) are important for Ca^2+^ regulation in neurons and the glial cells of the nervous system and have been also implicated in cytokine production and antigen response in immune cells [260]. The store-operated calcium entry (SOCE) channel *Orai3* is involved in LUAD cell proliferation, since *Orai3* can regulate cell cycle and cyclin expression [89].

#### 3.4.3. Stromal Interaction Molecule 1

Stromal interaction molecules (STIM) are transmembrane proteins that respond to Ca^2+^ fluctuations in the endoplasmic reticulum [261]. *STIM1* overexpression in HPV+ CC regulates the production of vascular endothelial growth factor A (VEGF-A), increasing the invasive capacity of cells. Likewise, its silencing arrests the cell cycle in the S and G_2_/M phases and increases the expression of p21 protein [58].

#### 3.4.4. Transient Receptor Potential Cation Channels

Transient receptor potential (TRP) channels act as cellular sensors of mechanical forces, taste, thermal sensation, and other physiological processes [262]. Several TRP subfamilies, including TRPC, TRPV, TRPM, TRPA, and TRPP, are suggested to participate in cancer [263]. 

The transient receptor potential cation channel subfamily C member 6 (*TRPC6*) is activated by the second messenger diacylglycerol (DAG) [264]. In human head and neck carcinoma, *TRPC6* is overexpressed in cancerous tissue samples in comparison to samples from non-cancer patients, and its inhibition significantly interferes with cell invasion, although it did not reduce cell proliferation [137]. *TRPV1* (transient receptor potential cation channel subfamily V member 1 or transient receptor potential vanilloid 1) is activated by nociceptive stimuli, especially regarding capsaicin, which is present in hot peppers and is a natural agonist of *TRPV1*; therefore, it is involved in pain perception, thermoregulation, and osmoregulation [262]. *TRPV1* is overexpressed in tongue cell carcinoma samples compared to healthy epithelium and is suggested as a tumor marker for this carcinoma [90]. However, vanilloids are cytotoxic in OSCC but are independent of *TRPV1* activation; hence, capsazepine was suggested as a therapeutic candidate for this cancer (Table 2) [138]. The transient receptor potential cation channel subfamily V member 2 (or transient receptor potential vanilloid 2, *TRPV2*) is mainly expressed in nerve, immune, and neuroendocrine cells, and it participates in osmoregulation and autonomic and cardiovascular modulation, as well as the regulation of the cytoskeleton and cell motility [265]. Its overexpression in ESCC patient tissue samples and cell lines is associated with cell cycle progression. *TRPV2* silencing leads to reduced cell proliferation, migration, and invasion, the induction of apoptosis, and an altered Wnt/β-catenin signaling pathway [139].

*TRPV3* (transient receptor potential cation channel subfamily V member 3 or transient receptor potential vanilloid 3) is mainly expressed in keratinocytes and is activated by repetitive thermal stimuli [262]. The overexpression of *TRPV3* in NSCLC is able to influence cell proliferation, channel blocking decreases the colony-forming capacity of cell lines, and the cell cycle is arrested in the G_1_/S phase (Table 2) [140]. The transient receptor potential cation channel subfamily V member 4 (or transient receptor potential vanilloid 4, *TRPV4*) is expressed in many tissues, is involved in the perception of various physical and chemical stimuli, and its expression has been associated with several neurodegenerative disorders [266]. In OSCC, their expression is higher in tumor samples than in the adjacent non-tumorous regions and is suggested to promote cell proliferation via AKT signaling calcium calmodulin kinase II (CAMKII) activation [91]. *TRPV6* (transient receptor potential cation channel subfamily V member 6 or transient receptor potential vanilloid 6) is expressed in the placenta, epidermis, digestive tract, and salivary glands, and its dysregulation is associated with several diseases, including cancer [267]. In ESCC, the low gene and protein expression of *TRPV6* is linked with the unfavorable survival of male patients but is favorable for women; thus, it may be regulated by sex steroid hormones [111].

The transient receptor potential cation channel subfamily M member 2 (or transient receptor potential melastatin 2, *TRPM2*) is associated with oxidative stress and has been related to cardiovascular and neurodegenerative disorders [262]. In some cancers, *TRPM2* has a protective role via reducing oxidative stress, inflammation, and chromosomal instability, although in other cancers it may promote tumor progression [268]. In OSCC, *TRPM2* is highly expressed in cell lines and cancerous samples from patients, and its knock-down by shRNA inhibits cell survival and migration; thus, the channel is proposed as an alternative target in the treatment of OSCC [141]. Likewise, in ESCC, the protein expression level of *TRPM2* is increased compared to normal tissue [142]. *TRPM7* (transient receptor potential cation channel subfamily M member 7 or transient receptor potential melastatin 7) is important in the intracellular regulation of magnesium (Mg^2+^), zinc (Zn^2+^), and Ca^2+^ concentration via the modulation of SOCE [269]. In head and neck carcinoma, a relevant Ca^2+^ current takes place via *TRPM7*, and its blocking or silencing reduces the growth and proliferation of cancer cells [270]. *TRPM7* is overexpressed in LUAD cell lines and samples from patients with the pathology and promotes the expression of stem cells markers. In addition, the reduction in gene expression by shRNA or channel block with waixenicin A inhibits cell viability (Table 2) [143]. The transient receptor potential cation channel subfamily A member 1 (or transient receptor potential ankyrin 1, *TRPA1*), which is activated by a wide range of irritant substances that can be found in food or in the environment, is mainly expressed in neuronal cells and has nociceptive functions [262]. In *SCLC*, *TRPA1* overexpression has been detected both in cell lines and patient tissue samples compared to non-malignant lung tissue, and its downregulation decreases the anchoring capacity of cancer cells [112].

*TRPP2* (polycystin 2 transient receptor potential cation channel or transient receptor potential polycystic receptor 2) is involved in mechanical sensation and cell proliferation [271]. In head and neck carcinoma cell lines, *TRPP2* silencing increases cell proliferation [144].

## 4. Conclusions

The high incidence of HPV-associated cancers causes thousands of deaths worldwide. Thus, novel diagnostic, prognostic, and therapeutic tools are urgently needed. The expression and regulation of ion channels in HPV-associated cancers here reviewed represents a plethora of opportunities and potential solutions to this problem. 

Because cancer is a multifactorial disease and ion channels are regulated by a variety of cancer-associated factors, more studies are needed to determine whether changes in ion channel expression are solely or mainly associated with HPV infection. For instance, an RNA-Seq database analysis of ion channel expression from HPV^+^ and HPV- tissues from the same patient or cancer type should be performed. This analysis should include information concerning the exposure of the patients to other cancer-associated factors, such as hormones, cigarette smoke, etc., to define the specific influence of each risk factor on ion- channel expression. It will be very interesting in a few decades to analyze ion channel expression in “HPV-associated cancers” in those patients who have been vaccinated against HPV. As mentioned above, this will help to resolve the controversy of the association of HPV infection with lung cancer and other malignancies. The investigation of more precise molecular mechanisms associating HPV-oncoproteins with the regulation of ion channels is necessary.

A significant percentage of drugs commonly used in clinical settings target ion channels. Glibenclamide and antihistamines are examples of the extraordinary opportunity that ion channel inhibitors may provide for drug repurposing for the benefit of cancer patients. The study of the combination of ion channel inhibitors with usually prescribed anticancer drugs represents an exceptional area for cancer research. This approach may help to reduce drug-resistance and fight cancer cells via diverse molecular mechanisms and cellular pathways. The vast amount of research yet to be performed associating ion channels and HPV could help to find novel markers and targets to improve clinical outcomes in patients with these types of malignancies.

## Figures and Tables

**Table 1:** HPV genetic types associated with the prevalence of different conditions.

Associated Cancer	Types	References
Invasive cervical cancer	HPV-16, HPV-18, HPV-45, HPV-31, HPV-33	[4]
High grade cervical precancerous lesions	HPV-16, HPV-31, HPV-52, HPV-18, HPV-58	[4]
Low grade cervical precancerous lesions	HPV-16, HPV-51, HPV-6, HPV-66, HPV-56, HPV-18	[4]
Normal cervical cytology	HPV-16, HPV-62, HPV-53, HPV-84, HPV-51	[4]
Anal cancer	Cancerous types HPV-16, HPV-33, HPV-31, HPV-58, HPV-39, HPV-52	[4]
	Non-cancerous types HPV-40, HPV-42, HPV-43, HPV-54, HPV-55	[4]
Anal precancerous infection in men	Cancerous types HPV-16, HPV-18, HPV-33, HPV-31, HPV-58	[4]
	Non-cancerous types HPV-6, HPV-74, HPV-11, HPV-44	[4]
Penile cancer	Cancerous types HPV-16, HPV-33, HPV-35, HPV-59, HPV-18	[4]
	Non-cancerous types HPV-6, HPV-11, HPV-32, HPV40, HPV-42	[4]
Vulvar cancer	Cancerous types HPV-16, HPV-33, HPV-18, HPV-45, HPV-52	[4]
	Non-cancerous types HPV-6, HPV-11, HPV-44, HPV-61	[4]
Vaginal cancer	Cancerous types HPV-16, HPV-31, HPV-33, HPV-18, HPV-52, HPV-73	[4]
	Non-cancerous types HPV-42, HPV-6	[4]
Oral cavity cancer	HPV-16, HPV-18, HPV-31, HPV-33, HPV-35	[4]
Oropharyngeal cancer	HPV-6, HPV-11, HPV-16, HPV-18, HPV-31	[4]
Laryngeal cancer	HPV-6, HPV-11, HPV-16, HPV-18, HPV-31	[4]
Lung cancer	HPV-6, HPV-11, HPV-16, HPV-18	[20]
Colorectal cancer	HPV-16, HPV-18	[21]
Bladder cancer	HPV-16, HPV-18, HPV-6	[22]
Breast cancer	HPV-18	[23,24]

**Table 2:** Compendium of ion channels and their potential clinical use in HPV-associated cancers.

Potential Clinical Use	Channel Family	Head and Neck Carcinoma ^1^	Cervical Cancer	Lung Cancer	References
**Diagnosis**	Potassium channels	*KCNH2 KCNQ1* *KCNJ15 KCNK3* *KCNK9 KCNK18*	*KCNH1* * KCNMA1 * KCNN4 * KCNJ11 *	*KCNQ* *KCNS3* *KCNJ4* *KCNJ8* *KCNK3*	[29,75,76,77,78,79,80,81,82,83,84,85,86]
	Calcium channels	*CACNA1G* *TRPV1* *TRPV4*	*STIM1* *	*CACNA1G CACNA1H* *Orai3*	[58,87,88,89,90,91]
	Sodium channels	*SCN2A* *SCN7A SCN5A*	*SCN8A* *	*SCN9A*	[31,92,93]
	Chloride channels	*ANO1* *CLCN2*		*ANO1*	[94,95,96,97,98,99,100,101]
**Prognosis**	Potassium channels	*KCNH2 KCNC4 KCNA3 KCNQ1OT1 KCNJ15*	KCNA1* KCNMA1*	*KCND2* *KCNQ1* *KCNAB2* *KCNN4* *KCNJ4*	[29,57,78,80,95,102,103,104,105,106,107,108,109]
	Calcium channels	*TRPV6*	*STIM1* *	*CACNA1B* *TRPA1*	[58,110,111,112]
	Sodium channels	*SCN5A SCNN1G SCN7A*	*SCN8A* *	SCN9A SCNN1B	[31,92,113,114,115,116]
	Chloride channels	*ANO1* VRAC * ANO9	*CLCA2*	*CLCA2* *CLIC1* *	[117,118,119,120,121,122,123]
**Therapy**	Potassium channels	*KCNH2* *KCNQ1OT1* *KCNMA1* *KCNN4*	*KCNH1* * KCNH2 * KCNC4 * KCNN4 * KCNJ11 *	*KCNH1* *KCNH2* *KCNA1* *KCNQ* *KCNS3* *KCNQ1OT1KCNAB2* *KCNN4* *KCNJ4* *KCNJ8* *KCNK3*	[56,72,74,75,76,77,82,83,104,124,125,126,127,128,129,130,131,132,133,134,135]
	Calcium channels	*CACNA2D3* *CACNA2D1* *TRPC6* *TRPV1* *TRPV2* *TRPM2* *TRPP2*	*STIM1*	*CACNA2D1* *CACNA1* *TRPV3* *TRPM7* *TRPA1*	[90,110,112,136,137,138,139,140,141,142,143,144]
	Sodium channels	*SCN5A SCNN1G*	*SCN8A* *	*SCN9A* *SCNN1A* *SCNN1B* *SCN5A*	[114,115,116,145,146,147,148,149]
	Chloride channels	*ANO1 ClC-3* VRAC * *ANO9 CLIC4*	*CIC-3*	*CLIC1* * *ANO1*	[46,51,94,96,98,99,100,101,117,118,119,120,150,151,152,153,154,155,156,157,158,159,160,161,162,163,164,165,166,167,168,169,170,171,172,173]

^1^ Includes tongue, oral, esophageal, laryngeal, and nasopharyngeal cancers. * HPV^+^ samples were studied, and channel expression level was compared with the corresponding normal cells or non-cancerous tissues.

## Data Availability

Not applicable.
